# Safety and efficacy of stenting for aortic arch hypoplasia in patients with coarctation of the aorta

**DOI:** 10.1007/s12471-019-01353-5

**Published:** 2019-11-29

**Authors:** E. G. Warmerdam, G. J. Krings, T. A. Meijs, A. C. Franken, B. W. Driesen, G. T. Sieswerda, F. J. Meijboom, P. A. F. Doevendans, M. M. C. Molenschot, M. Voskuil

**Affiliations:** 1 University Medical Center Utrecht, Utrecht, The Netherlands; 2 Netherlands Heart Institute, Utrecht, The Netherlands; 3 Central Military Hospital, Utrecht, The Netherlands

**Keywords:** Aortic coarctation, Hypertension, Stents

## Abstract

**Background:**

Despite a successful repair procedure for coarctation of the aorta (CoA), up to two-thirds of patients remain hypertensive. CoA is often seen in combination with abnormal aortic arch anatomy and morphology. This might be a substrate for persistent hypertension. Therefore, we performed endovascular aortic arch stent placement in patients with CoA and concomitant aortic arch hypoplasia or gothic arch morphology. The goal of this retrospective analysis was to investigate the safety and efficacy of aortic arch stenting.

**Methods:**

A retrospective analysis was performed in patients who underwent stenting of the aortic arch at the University Medical Center Utrecht. Measurements collected included office blood pressure, use of antihypertensive medication, invasive peak-to-peak systolic pressure over the arch, and aortic diameters on three-dimensional angiography. Data on follow-up were obtained at the date of most recent outpatient visit.

**Results:**

Twelve patients underwent stenting of the aortic arch. Mean follow-up duration was 14 ± 11 months. Mean peak-to-peak gradient across the arch decreased from 39 ± 13 mm Hg to 7 ± 8 mm Hg directly after stenting (*p* < 0.001). There were no major procedural complications. Mean systolic blood pressure decreased from 145 ± 16 mm Hg at baseline to 128 ± 9 mm Hg at latest follow-up (*p* = 0.014).

**Conclusion:**

This retrospective study shows that stenting of the aortic arch is successful when carried out in a state-of-the-art manner. A direct optimal angiographic and haemodynamic result was shown. No major complications occurred during or after the procedure. At short- to medium-term follow-up a decrease in mean systolic blood pressure was observed.

## What’s New

- Aortic arch stenting is a safe procedure.
- Aortic arch stenting results in a decrease in systolic blood pressure.
- Aortic arch stenting results in a decrease in need for antihypertensive medication.
- We elaborate on important procedural steps necessary for safe and successful stent placement.

## Introduction

Coarctation of the aorta (CoA) accounts for approximately 5–8% of all forms of congenital heart disease. It is characterised by a narrowing of the upper descending aorta, most commonly distal to the origin of the left subclavian artery near the insertion of the arterial ligament. CoA can occur as an isolated lesion, but frequently occurs in combination with other lesions such as a bicuspid aortic valve (50% of patients), ventricular septal defect (15% of patients), or a hypoplastic aortic arch (13% of patients) [1–4]. Clinical characteristics depend on the severity of the CoA and may vary from acute congestive heart failure in the neonate to systemic hypertension in late childhood or adulthood. Currently, percutaneous stent placement is the standard of care for adults and older children [5].

Even after successful CoA repair, residual or late-onset systemic hypertension is not uncommon. It is seen in up to two-thirds of the adult patients after an initial successful repair [6, 7]. Systemic hypertension contributes to a higher morbidity and mortality in these patients, due to an increased incidence of cerebrovascular diseases, heart failure, and acceleration of the progression of coronary artery disease [8]. Although successful stenting of native or recurrent CoA does not always eliminate the need for treatment with antihypertensive drugs, it can facilitate optimal medical treatment [9].

A substantial number of patients with CoA have an abnormal aortic arch anatomy or geometry. It is thought that neonatal aortic arch hypoplasia in CoA patients is caused by a decreased flow to the aorta in utero and that catch-up growth of the transverse aortic arch after repair is limited [10, 11]. Several studies have found that aortic arch hypoplasia is associated with late systemic hypertension [12, 13], even in the absence of an arm-leg blood pressure gradient [8].

The influence of abnormal aortic arch geometry on systemic hypertension is still a subject of debate. An abnormal aortic flow and increased aortic stiffness have been observed in aortic arches with gothic morphology [14]. Deviating morphology may lead to hypertension [15, 16]. Both systemic hypertension and abnormal blood pressure response to exercise have been reported in this population [17, 18]. A hypoplastic or gothic aortic arch might be a substrate for persistent hypertension, and stenting of the aortic arch could be beneficial for this patient cohort. The main objectives of this study were to investigate the safety of this treatment strategy and to investigate the effect on blood pressure regulation of aortic stenting in adolescent and adult patients.

## Methods

### Study population

A retrospective review of the cardiac interventional database at the University Medical Center Utrecht (Utrecht, the Netherlands) was performed to identify patients for this study. Patients were selected when they met the following inclusion criteria: (1) diagnosed with CoA; (2) undergone stent implantation that included the aortic arch; (3) a body weight >50 kg. Arch stenting was defined as stenting between the brachiocephalic trunk and the left subclavian artery. Due to the retrospective nature of this study, an ethics waiver was granted by the local Medical Ethical Committee.

### Data acquisition

Data on demography, biometry, blood pressure, medication use, cardiac imaging, and catheterisation procedures were collected from the electronic patient records. Data were collected at two different time points: before stent implantation and at the most recent outpatient visit.

### Measurements

The office blood pressure was measured in a seated position in the right upper limb with an automated cuff in accordance with the European Society of Cardiology (ESC) guideline [19].

The ascending and descending aortic pressure were measured during cardiac catheterisation for evaluation of the peak-to-peak systolic pressure over the arch. During catheterisation both two-dimensional angiograms and three-dimensional rotational angiograms were obtained, before and after stent implantation. In three-dimensional rotational angiography the measurements were performed using multi-planar reconstructions. The ascending aorta was measured just before the brachiocephalic trunk, the descending aorta was measured at the level of the diaphragm and the transverse aortic arch was measured at the narrowest diameter. In case the aorta had an ‘oval’ shape, surface area was calculated using the formula: *surface area* = *radius a ∗ radius b ∗ π*.

### Stent implantation technique

All procedures were performed under general anaesthesia. In all patients, vascular access was achieved using the right femoral artery. The DynaCT Artis Zee system (Siemens Healthcare, Erlangen, Germany) was used for performing three-dimensional rotational angiography. The three-dimensional reconstructions gathered with this system were used as an overlay over our fluoroscopy images for optimal procedure guidance, as previously published [20]. The decision to proceed to stent placement was made taking into account several parameters: the peak-to-peak gradient across the aortic arch, the presence of an anatomical substrate, the presence of collaterals, and the presence of hypertension in daily life (preferably confirmed with 24-hours ambulatory blood pressure measurements). Target stent diameter and length were determined based on three-dimensional rotational angiography measurements, conventional two-dimensional angiography measurements of dimensions of the ascending aorta just before the brachiocephalic trunk and the descending aorta at the level of the diaphragm, and balloon interrogation. In complex arch morphology a steerable long sheath (Oscor, 12–13,8 French) as well as rapid pacing were used. Mainly, ev3 Max LD (Medtronic, Plymouth, MN, USA), Andra XXL (Andramed GmbH, Reutlingen, Germany) and Cheatham-Platinum (CP) stent (NuMED Inc., Hopkinton, NY, USA) were used. Strut dilatation to side branches was performed when deemed necessary to enhance left carotid or left subclavian flow. In selected patients with complex aortic morphology two procedures were planned. Stents were placed in the first procedure and consequently dilated further in the second procedure. Major complications were defined as stroke, myocardial infarction, bleeding classified as BARC >2 (Bleeding Academic Research Consortium scale), or death.

### Data analysis

All analyses were performed using SPSS statistical software version 25 (IBM SPSS Data Collection, Chicago, IL, USA). Descriptive statistics were used for demographic data. Quantitative data are presented as mean ± standard deviation or absolute number (percentage). Group means before and after stent placement were compared using the paired samples t‑test. Results were considered statistically significant if the probability value (*p*-value) did not exceed 0.05.

## Results

### Demographic data

Between April 2014 and January 2018 a total of 12 patients with a mean age of 24 ± 8 years underwent stenting for aortic arch hypoplasia or gothic arch morphology. Eleven patients previously had some form of CoA repair, one patient had a native CoA. Eleven patients had a hypoplastic aortic arch, one patient had a gothic arch morphology. Ten patients had concomitant congenital cardiac defects. Follow-up data were available for all patients; mean follow-up duration was 14 ± 11 months. Patient characteristics are presented in Tab. 1.

**Table 1:** Baseline characteristics

Variable	Patients (*n* = 12)
Age (years)	24 ± 8
Male	9 (75%)
Weight (kg)	70 ± 7
BMI (kg/m^2^)	23 ± 2
Native CoA	1 (8%)
*Concomitant cardiac defects*	
– Bicuspid aortic valve	6 (50%)
– Ventricular septal defect	4 (33%)
– Persistent ductus arteriosus	2 (17%)
– Transposition of the great arteries	1 (8%)
*Previous CoA repair*	
– End-to-end anastomosis	7 (58%)
– Patch angioplasty	4 (33%)
– Balloon dilatation	3 (25%)
– Stent implantation	5 (42%)
*Medication use*	
– ACE inhibitor	4 (33%)
– Angiotensin II receptor blocker	4 (33%)
– Beta-blocker	1 (8%)
– Calcium channel blocker	4 (33%)
– Diuretics	3 (25%)

Data are presented as number (percentage) or mean with standard deviation (±)

*BMI* body mass index, *CoA* coarctation of the aorta, *ACE* angiotensin-converting enzyme inhibitor

### Procedural data

Femoral artery sheath sizes ranged from 8–14 French. During the stenting procedure 21 stents were used in a total of 12 patients: the CP stent was used in 6 (50%) patients, the ev3 Max LD stent was used in 5 (42%) patients, the ev3 Mega LD stent was used in 3 (25%) patients, and the Andra XXL stent was used in 1 (8%) patient. The length of the used stents varied from 26–57 mm. After stent implantation, post-dilatation of the stent was performed in 10 patients using the Atlas PTA Balloon (Bard Peripheral Vascular, Tempe, AZ, USA) in six (50%) patients and the Cristal balloon (ab medica, Dusseldorf, Germany) in four (33%) patients, with balloon inflation pressures ranging between 10–24 atm. Aortic arch vessels were crossed in all patients; in six patients the left subclavian artery was crossed, in two patients the left common carotid artery was crossed, and in four patients both the left subclavian artery and the left common carotid artery were crossed.

### Acute angiographic result

**Fig. 1:**
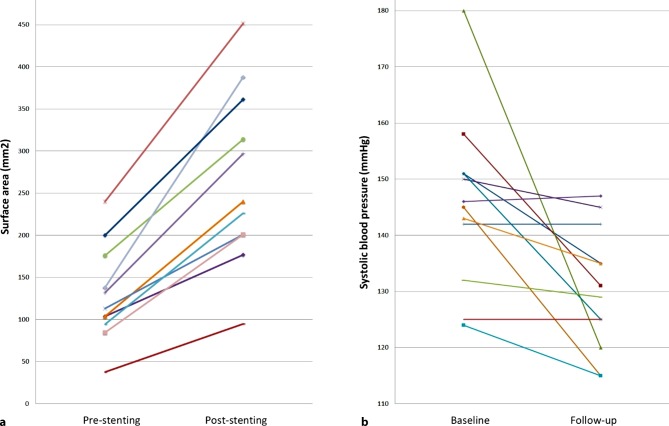
Result of stenting on surface area and systolic blood pressure. **a** Surface area (mm^2^) as measured on three-dimensional rotational angiography multiplanar reconstructions pre- and post-stenting. **b** Systolic blood pressure (mm Hg) measured at the right arm at baseline and follow-up

The mean peak-to-peak gradient across the aortic arch decreased from 39 ± 13 mm Hg to 7 ± 8 mm Hg after stent placement (*p* < 0.001). The mean orthogonal diameters at the narrowest point of the transverse aortic arch increased from 12 ± 3 mm × 13 ± 3 mm to 18 ± 3 mm × 19 ± 4 mm after stent placement (*p* < 0.001 and *p* < 0.001 respectively). Resulting in an increase in mean surface area of 126 ± 56 mm^2^ to 276 ± 107 mm^2^ (*p* < 0.001). Data are presented in Tab. 2 and Fig. 1.

**Table 2:** Acute angiographic results

	Pre	Post	*p*-value
PG (mm Hg)	39 ± 13	7 ± 8	<0.001
Aortic arch narrowest point			
– Sagittal diameter (mm)	12 ± 3	18 ± 3	<0.001
– Corresponding orthogonal diameter (mm)	13 ± 3	19 ± 4	<0.001
– Surface area (mm^2^)	126 ± 56	276 ± 107	<0.001
Descending aorta caudal			
– Sagittal diameter (mm)	18 ± 5	NA	NA
– Corresponding orthogonal diameter (mm)	18 ± 5	NA	NA
– Surface area (mm^2^)	716 ± 472	NA	NA

Orthogonal diameters were measured using three-dimensional rotational angiography. Data are presented as mean with standard deviation (±)

*NA* not applicable, *PG* peak gradient measured over the aortic arch during catheterization

### Procedural complications

No major complications occurred during the procedure or follow-up, we observed three minor complications. A temporary third-degree atrioventricular block occurred in one patient. During the post-implantation redilatation of one of the stents a stent fracture occurred in one patient, which was resolved by placement of a covered stent. One patient experienced minor rebleeding of the access site, which was managed with a simple bandage. No endovascular leaks occurred after stent implantation.

### Follow-up

Four patients underwent a planned staged procedure, with successful further dilatation of the stent in the first year after the implantation. Data presented are at baseline (before stent implantation) and at latest follow-up (after further dilatation). No major complications occurred during mean follow-up of 14 ± 11 months.

### Blood pressure regulation

There was a decrease in mean systolic blood pressure, measured at the right arm, from 145 ± 16 mm Hg at baseline to 128 ± 9 at latest outpatient visit (*p* = 0.014) (Fig. 1). There was no significant decrease in mean diastolic blood pressure (*p* = 0.477). A decrease in the mean number of antihypertensive drug classes used was observed from 1 ± 1 before stent placement to 0 ± 1 at the most recent outpatient visit (*p* = 0.016). Data on blood pressure and antihypertensive medication for each individual patient are presented in Tab. 3.

**Table 3:** Blood pressure and medication

Patient no	BP baseline (mm Hg)	AHD baseline	BP post stent (mm Hg)	AHD post stent
1	151/98	None	135/63	None
2	158/60	Losartan 50 mg Metoprolol 25 mg	131/63	None
3	180/95	Verapamil 240 mg	120/91	None
4	150/74	None	145/85	None
5	151/71	Telmisartan 80 mg	125/75	None
6	145/90	None	115/70	None
7	142/80	Lisinopril 20 mg	142/77	None
8	125/80	Telmisartan 40 mg Amlodipine 5 mg	125/70	Telmisartan 40 mg Amlodipine 5 mg
9	124/57	None	129/69	None
10	124/56	Lercanidipine 5 mg Lisinopril 20 mg Hydrochlorothiazide 25 mg	115/57	Lisinopril 20 mg
11	146/55	Ramipril 10 mg	147/68	Ramipril 10 mg
12	143/67	Olmesartan 40 mg Amlodipine 10 mg Hydrochlorothiazide 25 mg	135/70	Olmesartan 40 mg Amlodipine 10 mg Hydrochlorothiazide 25 mg

Data on blood pressure and antihypertensive medication for each patient before stent placement and at latest follow-up after stent implantation

*AHD* antihypertensive drugs, *BP* blood pressure, *no* number

## Discussion

In this retrospective study we analysed a subset of patients with CoA and a concomitant hypoplastic or gothic aortic arch who underwent stenting of the aortic arch. Stenting of the aortic arch was successful in all selected patients and no complications occurred. The acute angiographic result was excellent, demonstrated by an increase in aortic arch surface area and a decrease in mean peak-to-peak gradient across the aortic arch. Most importantly, a significant decrease in mean systolic blood pressure was observed, with a concomitant decrease in the need for antihypertensive medication.

The target of interventional treatment in CoA is the relief of the obstruction and reduction of the pressure gradient. A large number of patients still suffer from residual or late-onset systemic hypertension despite successful initial percutaneous or surgical therapy. A substantial number of patients who have a CoA also have an abnormal aortic arch anatomy or morphology, which might be a substrate for hypertension. Percutaneous stent placement has become the standard of care for the treatment of CoA after childhood in the last decade. However, stent placement as treatment for aortic arch hypoplasia has only been described in small series and case reports [21–25]. All reports on aortic arch stenting showed anatomical and physiological relief of obstruction. Boshoff et al. reported no peri-procedural complications [24], Holzer et al. reported a relatively high number of adverse events (in 31% of patients), although this was mostly in patients with a weight below 10 kg or with univentricular physiology [25]. In a study including 21 patients, Pushparajah et al. reported three major complications in two patients [21]. Stent migration occurred in two patients, of which one subsequently suffered from an embolic stroke. In these two cases stents were implanted without the use of rapid pacing, which was thought to have increased the risk of stent migration. In our cohort no major complications were observed peri-procedurally or during follow-up.

**Fig. 2:**
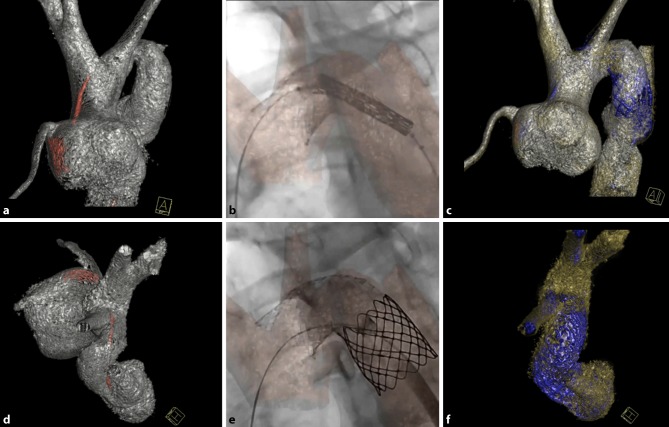
Stent implantation in gothic aortic arch after arterial switch operation. A 36-year-old male with a history of dextro-transposition of the great arteries and coarctation of the aorta. He presented with persisting hypertension late after arterial switch operation. Three ev3 Mega LD stents and one non-covered CP stent were implanted. **a**,**c**,**d**,**f** Three-dimensional reconstructions made from three-dimensional angiography data. **a** Anterior view before stent implantation. **d** Cranial view before stent implantation. **b** and **e** Conventional two-dimensional fluoroscopy images showing stent implantation. **c** Anterior view after stent implantation. **f** Cranial view after stent implantation

**Fig. 3:**
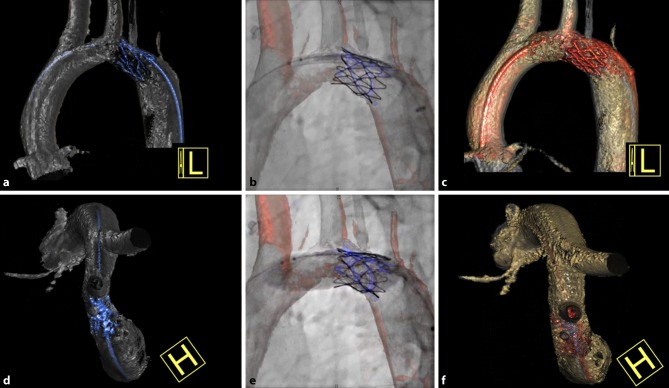
Stent implantation in hypoplastic aortic arch. An 18-year-old female with a history of coarctation of the aorta, for which she underwent surgical coarctation repair (end-to-end anastomosis) as an infant. At the age of seven a CP stent was implanted for re-coarctation. Since she remained hypertensive in the presence of a narrow aortic arch, an ev3 Mega LD stent was implanted in the aortic arch. **a**,**c**,**d**,**f** Three-dimensional reconstructions made from three-dimensional angiography data. **a** Lateral view before stent implantation. **d** Cranial view before stent implantation. **b** and **e** Conventional two-dimensional fluoroscopy images showing stent implantation. **c** Lateral view after stent implantation. **f** Cranial view after stent implantation

We believe several steps are important for a safe and successful procedure. Understanding the aortic arch anatomy and morphology is vital, cardiac magnetic resonance imaging (MRI) and computed tomography (CT) are therefore essential in the pre-procedural phase. During the procedure we use reconstructions derived from three-dimensional rotational angiography as an overlay on our fluoroscopy images for optimal guidance (see Fig. 2 and 3). Balloon interrogation can provide information regarding correct size, tissue compliance and balloon stability. Rapid pacing and deployment of the stent through a steerable long sheath can enhance positioning effectively and reduce the risk of stent migration. A stent with moderate radial strength and good compliance—such as the ev3 Max LD and ev3 Mega LD—serves best to achieve an ideal aortic arch shape. True open cell design is mandatory to enable strut dilatation when necessary to enhance carotid or subclavian flow. Typically, we aim to position an arch stent between the brachiocephalic trunk and the left subclavian artery with the proximal and distal part reaching out into the ostia. We believe that this technique results in an optimal distribution of shear stress to reduce intimal trauma of the aortic arch.

Pushparajah et al. investigated the effect of stent implantation on blood pressure and antihypertensive medication at short- and medium-term follow-up. An improvement in blood pressure outcome was seen, with a decrease in median systolic blood pressure from 145 to 128 mm Hg. Antihypertensive medication could be reduced in 13 out of 17 patients. These results are in conjunction with our results on blood pressure regulation. Even though the goal of stent placement in the aortic arch is not to stop medical therapy, a decrease in antihypertensive medication is beneficial for this patient population. For these relatively young patients lifelong use of multiple medications is very demanding. Therefore, optimal treatment of the underlying substrate seems a sensible approach.

Several mechanisms are thought to increase the risk of systemic hypertension after successful CoA repair. It is hypothesised that these patients have an abnormal baroreceptor function and a decreased aortic compliance. Furthermore, age at initial CoA repair is known to be of great influence on the risk of long-term hypertension [26]. A hypoplastic or gothic aortic arch morphology might also be a substrate for persistent hypertension [11, 12, 16, 17]. The use of three-dimensional rotational angiography changed our understanding of the aortic arch anatomy and helped to accurately diagnose aortic arch hypoplasia and gothic morphology. Biplane angiographic projections as well as lateral and frontal views are not always sufficient; cranial and posterior views are typically essential to detect aortic arch hypoplasia. Such views can only be obtained from pre-procedural CT angiogram or MRI or peri-procedural three-dimensional rotational angiography.

Hypertension after CoA repair is not a benign condition and should be treated, regardless of its aetiology. Strict follow-up using advanced three-dimensional imaging and timely invasive haemodynamic evaluation and, if deemed necessary, intervention/reintervention is important in this patient group. When aortic arch hypoplasia is thought to play an important role in the presence of persistent hypertension, stent implantation should be considered to improve clinical outcome in the long term.

## Limitations

First, due to the retrospective nature of the study there was no clear protocol for the measurements before and after stent implantation, which resulted in missing data. Second, the population size of this study was quite small. Third, very incomplete data on 24-hour ambulatory blood pressure measurements were available. It is well known that this is a more reliable technique than office blood pressure measurement and gives a more comprehensive assessment of the patient’s blood pressure [27, 28]. Finally, although blood pressure response to exercise would have been an interesting parameter to examine, only a limited number of patients underwent exercise testing. Data on blood pressure response to exercise were therefore omitted.

## Conclusion

The present analysis shows that stenting of the aortic arch is successful when carried out in a state-of-the-art manner. It may lead to improved clinical outcome for this specific patient subset with abnormal aortic arch anatomy or morphology. Stent placement in our cohort achieved a direct optimal angiographic and haemodynamic result. No major complications occurred during or after the procedure. At short- to medium-term follow-up a significant decrease in systolic blood pressure was observed, combined with a parallel decrease in the use of antihypertensive medication.
